# Spatial Association of Food Sales in Supermarkets with the Mean BMI of Young Men: An Ecological Study

**DOI:** 10.3390/nu11030579

**Published:** 2019-03-08

**Authors:** Sabine Güsewell, Joël Floris, Claudia Berlin, Marcel Zwahlen, Frank Rühli, Nicole Bender, Kaspar Staub

**Affiliations:** 1 Institute of Evolutionary Medicine, University of Zurich, Winterthurerstrasse 190, CH-8057 Zurich, Switzerland; sabine.guesewell@iem.uzh.ch (S.G.); joel.floris@iem.uzh.ch (J.F.); frank.ruehli@iem.uzh.ch (F.R.); nicole.bender@iem.uzh.ch (N.B.); 2 Institute of Social and Preventive Medicine, University of Bern, Mittelstrasse 43, CH-3012 Bern, Switzerland; claudia.berlin@ispm.unibe.ch (C.B.); marcel.zwahlen@ispm.unibe.ch (M.Z.)

**Keywords:** cultural language regions, dietary patterns, food purchases, healthy food balance, obesity, socioeconomic status, urban vs. rural

## Abstract

Supermarket food sales data might serve as a simple indicator of population-level dietary habits that influence the prevalence of excess weight in local environments. To test this possibility, we investigated how variation in store-level food sales composition across Switzerland is associated with the mean Body Mass Index (BMI) of young men (Swiss Army conscripts) living near the stores. We obtained data on annual food sales (2011) for 553 stores from the largest supermarket chain in Switzerland, identified foods commonly regarded as “healthy” or “unhealthy” based on nutrient content, and determined their contribution to each store’s total sales (Swiss francs). We found that the sales percentages of both “healthy” and “unhealthy” food types varied by 2- to 3-fold among stores. Their balance ranged from −15.3% to 18.0% of total sales; it was positively associated with area-based socioeconomic position (*r* = 0.63) and negatively associated with the mean BMI of young men in the area (*r* = −0.42). Thus, even though we compared supermarkets from a single chain, different shopping behaviors of customers caused stores in privileged areas to sell relatively more healthy food. Knowledge about such patterns could help in designing in-store interventions for healthier nutrition and monitoring their effects over time.

## 1. Introduction

The prevalence of excess weight (overweight and obesity) in modern societies varies considerably among and within countries [1]. This spatial variation is related to socioeconomic factors such as the average income and education of the resident population [2,3,4]. The resulting spatial association of excess weight prevalence with socioeconomic status is partially mediated by variation in dietary quality [3,4,5]. Therefore, subpopulations with low dietary quality should be the primary target of public health measures against obesity [4]. Setting such priorities requires simple and reliable indicators of population-level diet composition [6]. There is still a need to identify suitable indicators and to confirm their associations with excess weight prevalence.

Easy access to supermarkets was found to be associated with a healthier diet and a lower prevalence of excess weight in a number of studies [7,8,9,10,11,12,13,14,15]. These associations have been explained by the greater choice and affordability of healthy foods available in supermarkets, as opposed to that within alternative food outlets, such as convenience stores [16]. Thus, the spatial distribution of supermarkets in a region provides some information about spatial patterns in population-level diet quality [11,15]. However, since supermarkets offer a wide range of food types, the actual composition of food purchases made in them depends on consumer choices [17]. Thus, variation in purchasing behavior within supermarkets is likely to better reflect variation in diet quality than just supermarket presence. Testing this hypothesis requires detailed, precise and representative data for multiple small spatial units across a large region. Such data could not be generated through traditional approaches, such as individual-level questionnaires or purchase diaries, because the sampling effort would have been prohibitive [6].

An efficient new approach was made possible by modern checkout systems, which automatically record all food sales made by a store. These store-level sales represent the aggregated purchasing choices of the store’s customers, many of whom usually live in the neighborhood. Sales from multiple stores with identical product offer can therefore reveal spatial patterns in the purchasing behavior of populations living at different places. Comparative studies have suggested that store-level sales data are indeed indicative of regional nutritional patterns [18,19,20], but their potential has only rarely been exploited so far. Using this approach, Howard Wilsher et al. [18] showed that store-based annual sales of unhealthy foods relative to healthy foods in a large UK supermarket chain were spatially related to the prevalence of excess weight among children. It still remains to be shown whether such an association also exists for adults and in Western European countries with a lower prevalence of excess weight and higher income levels than the UK [21]. 

We explored these questions for Switzerland, a high-income country with three culturally distinct regions determined by the spoken language. Earlier studies have shown that both the prevalence of excess weight [22,23,24,25] and nutrition patterns differ among these large regions [6,26,27,28]. Spatial associations between excess weight and nutrition patterns have not yet been explored at a smaller scale (e.g., differences between urban centers and rural areas) due to a lack of sufficiently detailed and representative data [29].

We therefore used annual food sales of the largest Swiss supermarket chain as a proxy for spatial patterns in population-level food purchases across Switzerland. We combined these sales data with standardized Body Mass Index (BMI) measurements on young men recruited by the Swiss Army (conscripts) living around each store as a proxy for excess weight prevalence in the young adult population. We investigated three sets of questions. First, how much do sales of “healthy” and “unhealthy” food types vary among stores of a single supermarket chain? How are these variations related to each other, and how are they related to patterns in sales of other commonly consumed food types? Second, does the balance of healthy vs. unhealthy food sales depend on the socioeconomic position (SEP) of the stores’ neighborhood, on language region and on urbanicity? Third, is the balance of healthy vs. unhealthy food sales related to the mean BMI of young men living the stores’ neighborhood? If so, is this relationship uniform or does it vary among language regions or between urban and rural areas? 

## 2. Materials and Methods

### 2.1. Food Sales Data

Food sales data from all stores (*n* = 687) belonging to one of the largest Swiss supermarket chains (market share in 2011: 37.7% for food products [30]) were obtained for the year 2011 [31]. Our analysis was based on the 553 supermarkets selling food for home consumption that were located in Swiss residential areas. We thus excluded restaurants, catering services, take-aways, non-food stores, independent retailers selling the chain’s products (with a different sales pattern related to small store size), shopping malls in purely industrial areas (selling to non-resident customers) and stores located in Liechtenstein. According to the chain’s information, all supermarkets follow an identical framework of product offers, with minor variations at the individual store level in response to customer demand. 

**Figure 1:**
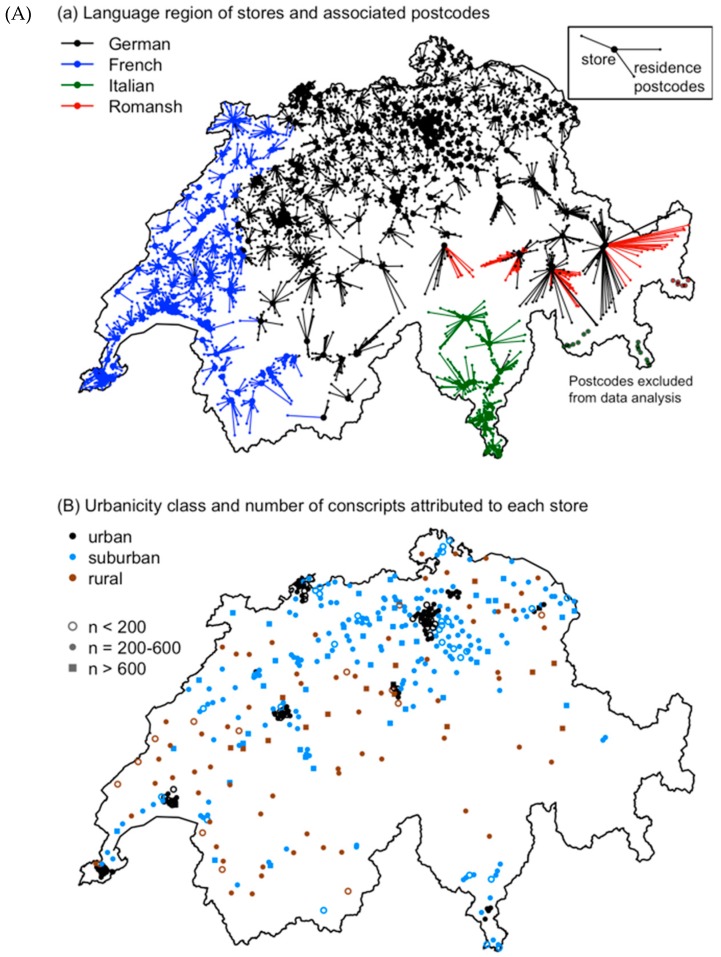
Distribution of stores (points) (**A**) with lines indicating the postcodes of residence attributed to each store based on spatial proximity and colors showing language regions and (**B**) with urbanicity class and the number of young men with BMI data (Swiss Army conscripts) attributed to each store.

The 553 stores were located in 445 distinct postcode-defined areas (hereafter called “postcodes”), because 53 postcodes included two stores, and 22 included 3–6 stores. Stores within the same postcode were combined by adding their sales. These postcode-level units will be called “stores” hereafter for simplicity. The spatial distribution of stores is shown in Figure 1, and store numbers for each language region and degree of urbanization are given in Table 1.

Food sales data were provided by the supermarket chain as the total annual (2011) volume of sales in Swiss Francs (CHF) for many product categories that were classified according to a hierarchical system used specifically by this company for their logistics. For our analysis, we defined 12 food types at various levels of the classification, such that most foods included in one group would be commonly regarded as either “unhealthy” or “healthy” or indicative of a health-oriented lifestyle (Table 2). Foods classified as “unhealthy” are energy-dense and contain much sugar, fat and/or salt, so that their intake should be limited to avoid weight gain, whereas foods classified as “healthy” are rich in proteins, fibers, minerals and/or vitamins, and should be consumed regularly according to dietary guidelines. For the sake of simplicity, we use these category names without quotes in the remainder of the paper. We further identified two food types with ambivalent nutritional status and several broader basic food categories representing a large fraction of most Swiss people’s diets, which could not be categorized according to nutritional value with the available product classification (Table 2). Alcohol and tobacco do not appear in our analysis because they are not sold by this supermarket chain.

### 2.2. BMI of Swiss Conscripts

We obtained a representative dataset for the BMI values of all Swiss young men using the data collected in the recruitment process of the Swiss Army, as described in detail elsewhere [22,23,32]. In short, all men with Swiss nationality are examined for recruitment during the year in which they turn 19 unless they suffer from a severe disease or severe physical or psychiatric disability. The military assessments include, among others, standardized measurements of height (rounded to the nearest cm) and weight (rounded to the nearest kg), both measured without shoes in light underwear. Earlier studies estimated that these assessments cover >90% of the Swiss male population [23]. The medical causes for exemption are diverse and mostly not causally linked to body height or weight, and they are therefore unlikely to cause a bias in the data.

As a basis for this study, the Swiss Armed Forces provided a complete set of anonymized conscript records from 1 January 2010 to 31 December 2015 [23,33]. The data used here include the categorized age at conscription, BMI (kg/m^2^), and the postcode of the place of residence. Most conscripts (73.3%) were between 18.5 and 20.5 years old (the regular age), and we restricted the analyses presented here to this age group. 

### 2.3. Combination of Food Sales and BMI Data

Conscripts were attributed to stores based on their postcode of residence. Postcodes without a store were assigned to the nearest store using a Euclidean distance matrix based on spatial coordinates previously determined by the median population center within each postcode [22]. Assignments of residential postcodes to stores are shown in Figure 1A. Because there was no store in any of the Italian- or Romansh-speaking southeastern Alpine valleys (oriented towards Italy) nor in the adjacent central-Alpine Engadine valley, the southeastern valleys were excluded from this analysis (Figure 1A). In addition, some postcodes within the Alps were manually re-assigned to a different store, which was easier to reach or located in a culturally more similar region than the nearest store. There are no stores in the scattered northern-Alpine Romansh-speaking areas, so all Romansh speaking postcodes were assigned to stores in German-speaking areas (Figure 1A). At the German–French language border, some postcodes were also assigned to stores with a different language. In both cases, the transition between language regions is gradual, i.e., municipalities at the language border are generally bilingual, so a mixed-language assignment reflects the actual situation. 

The median number of conscripts assigned to a store was 294, with a range from 21 to 1179 (mean ± SD = 371.5 ± 197.5). The number of conscripts assigned to an individual store (cf. symbols in Figure 1B) was small in some sparsely populated areas, in regions with high store density around Zurich, and in some areas with many foreign residents in larger cities.

As we were interested in population-level spatial associations of BMI with food sales, we summarized individual conscript data using the mean BMI for each store. These store-level BMI data were then combined with the corresponding food sales data. As shown in Table 1, the mean store-level BMI was slightly higher in German- and Italian-speaking regions than in French-speaking regions, and it was slightly lower in urban municipalities than in rural ones.

### 2.4. Area-Based Socioeconomic Position (Mean Neighbourhood Swiss-SEP) and Urbanicity

The mean Swiss-SEP 2.0 index was calculated at postcode level on a scale from 0 to 100 [34]. The original Swiss neighborhood index of socioeconomic position (Swiss-SEP 1.0) was constructed using georeferenced census and road network connectivity data to calculate a household-centered index based on the neighborhood’s median flat rent per m^2^ and mean number of persons per room (two proxies for income and living standard), the proportion of households headed by a person with primary education or less, and the proportion headed by a person in a manual or unskilled occupation [34]. The Swiss-SEP 2.0 was based on population censuses and the 2012–2015 Swiss structural surveys, which were questionnaires completed by a random sample of about 250,000 persons. The Swiss-SEP 2.0 was constructed using the same methodology as the Swiss-SEP 1.0 with minor adaptions as the head of household was not defined. For each store, the mean SSEP was calculated as the mean SSEP-value of all conscripts assigned to that store. As shown in Table 1, the mean SSEP was substantially higher in urban areas than in rural areas, and it was slightly higher in the German-speaking region than in French- and Italian-speaking regions.

The urbanicity of each store’s municipality in 2011 was taken from the “Municipality Typology” published by the Swiss Federal Statistical Office (updated version from 1 January 2013). We used the Eurostat DEGURBA (degree of urbanization) classification based on population density [35]. For easier reading, we labelled the three DEGURBA categories as “urban” (densely populated area), “suburban” (intermediate density area) and “rural” (thinly populated area). The urbanicity class assigned to each store is shown in Figure 1B. 

### 2.5. Data Analysis

Annual sales of each food type in each store were expressed as the percentage of each store’s total annual food sales. Sales of the six healthy and the six unhealthy food types were summarized by calculating the sum of percentages for each group. The difference between these two percentages, hereafter called the “healthy sales balance” (HSB), was used to measure the extent to which healthy food sales exceeded unhealthy ones, with negative values indicating excess unhealthy sales. This measure was suitable because the mean sales of the two groups were almost identical (20.5% vs. 21.2%), and their sum was relatively constant, as it ranged from 38% to 48% in 98% of the stores.

To confirm the existence of a single healthy–unhealthy food sales gradient and to explore its association with sales of other food types, we calculated a correlation matrix with Pearson’s *r* for all food type pairs based on percentage sales. This matrix was subjected to principal components analysis (PCA) to identify the main axes of variation in the sales of the 20 pre-defined food types (Table 2) and the contribution of each food type to each PCA axis (loading). PCA was performed with the function “prcomp” in the software R, version 3.5.1 (R Core Team, Vienna, Austria, 2018) [36]. This R version was also used for all analyses described hereafter.

To analyze how the healthy sales balance (HSB) depends on the area-based SEP, language region and urbanicity class, we fitted linear models for the three factors separately and in combination (multiple regression) and present adjusted r-squared values of these models as measures of explained variation. We focused on models using only the main effects of the predictors after checking that little additional variation (<2%) was explained by including interaction terms. The relationship of the mean BMI of conscripts with the HSB (categorized into quartiles for easier interpretation) was analyzed similarly with a simple regression and with a multiple regression that included the area-based SEP, language region and urbanicity as possible confounders.

For both the HSB and the mean BMI, we examined to what extent spatial patterns across Switzerland are accounted for by the multiple regression models. We created point maps of raw data and adjusted data, calculated as the overall mean plus the residuals of the multiple regression. To see whether the residual spatial autocorrelation influenced our regression results, we included a spherical spatial correlation structure into the multiple regression models using generalized least-squares fitting (function “gls” in R package “nlme”) and compared the coefficients of these models to those from the ordinary least-squares regression.

### 2.6. Ethics

The BMI data were provided by the Swiss Armed Forces upon submission and approval of a study protocol. According to the Swiss federal law (Bundesgesetz über die militärischen Informationssysteme MIG, BG 510.91, Art. 2, 9, 24–29), the Swiss Armed Forces are authorized to make these data accessible in fully anonymous form for academic research. Thus, our data included residential postcodes but no exact addresses. Because Swiss conscription is mandatory, and our analyses only used anonymized, nonclinical, governmental data, no informed consent and no additional ethical approval was required (Swiss data privacy act, SR 235.1; 19.6.1992 and Federal Act on Research involving Human Beings HRA, 810.30; 1.1.2014) [23].

### 2.7. Availability of Data und Material

The datasets analyzed during the current study are not publicly available due to data protection reasons, but they are available from the data owners Migros (Limmatstrasse 152, CH-8031 Zürich, Switzerland) and the Medical Service of the Swiss Armed Forces (A Stab—Sanität, Militärärztlicher Dienst, Worblentalstrasse 36, CH-3063 Ittigen, Switzerland) upon request and approval of a study protocol. 

## 3. Results

### 3.1. Patterns in Food Sales

The 20 food types included in our analysis represented, on average, 73.4% of a store’s total food sales volume (Table 2). Healthy food types represented, on average, 20.5%, and unhealthy food types represented 21.2%. Mean percentage sales for individual food types ranged from 0.1% to 16.3%. Standard deviations were typically small (0.05% to 1.53%), but total ranges varied by two- to ten-fold, showing considerable differences in the sales composition among the 445 stores (Table 2).

**Figure 2:**
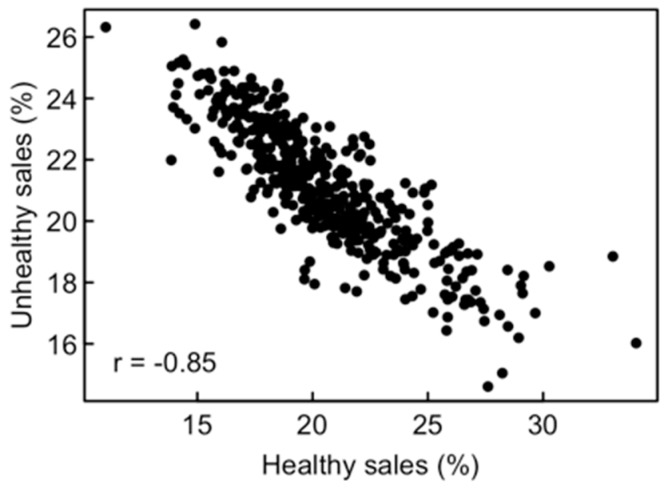
Negative correlations between sales of healthy and unhealthy food types across the 445 stores. Each point represents one store. Values are the sum of sales of the six food types included in each group, expressed as the percentage of a store’s total annual food sales.

Percentage sales of the six healthy food types mostly correlated positively with each other across stores (Figure S1 in Supplementary Materials; mean correlation coefficient *r* = 0.297). Sales of the six unhealthy food types also often correlated positively with each other (mean *r* = 0.171), whereas sales of most pairs of healthy and unhealthy food types were negatively correlated (mean *r* = −0.315). Due to these associations, the total percentage sales of the two groups varied widely among stores (healthy sales from 11.0% to 34.0%; unhealthy sales from 14.6% to 26.4%) and were strongly negatively correlated with each other (*r* = −0.85, Figure 2). Thus, the healthy sales balance (HSB), which ranged from −15.3% to 18.0%, summarized most of the variation in both healthy and unhealthy food sales among stores.

Principal components analysis (PCA) of the 20 food types also revealed a healthy-to-unhealthy sales gradient as the main axis of variation, which accounted for 25.4% of the total variation in percentage sales of the 20 food types. All healthy food types were negatively associated with the first PCA axis, and most unhealthy food types were positively associated with it (Table 3). Of the remaining food types, fruit juices, breakfast cereals, poultry and eggs were associated with healthy food sales, while pasta and milk products were associated with unhealthy food sales (Table 3). The second PCA axis (16.7% of variation) was negatively associated with sales of fruit and vegetables, bread and organic food (vegetarian diet), and was positively associated with sales of all types of meat and fish (Table 3). The third PCA axis (12.3% of variation) was negatively associated with milk products, meat and vegetables (low-carb diet), and was positively associated with sales of chocolate, cakes and bread (Table 3). All other PCA axes explained less than 10% of variation.

### 3.2. Factors Influencing Patterns in Food Sales

**Figure 3:**
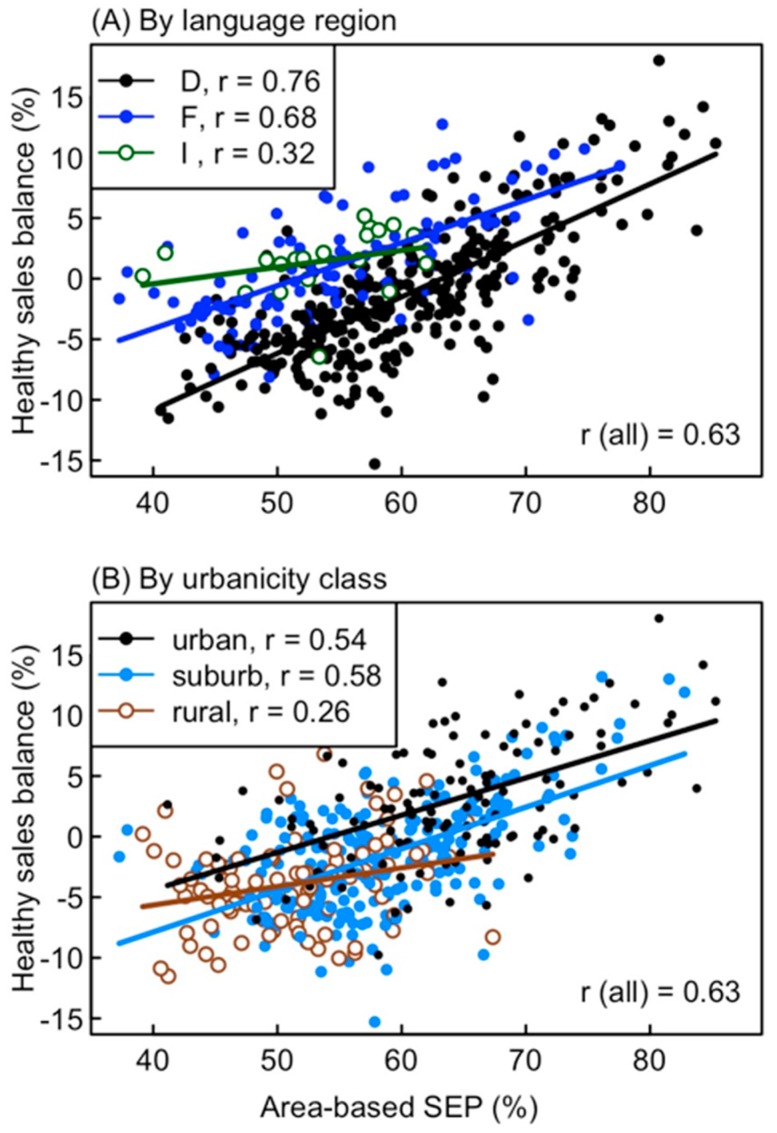
Healthy sales balance (HSB) of the 445 stores in relation to the area-based SEP (**A**) by language region (D = German, F = French, I = Italian) and (**B**) by urbanicity class. Pearson correlation coefficients and ordinary regression lines are given for each subset of stores, and correlation coefficients are also given for all stores together. HSB is the difference between healthy and unhealthy sales as the percentage of total food sales. Confidence intervals for correlation coefficients are given in Table S1.

The HSB of stores strongly depended on the area-based SEP, language region and urbanicity class. In simple regression models, these three predictors respectively explained 39.1%, 5.0%, and 23.4% of the variation in HSB. In a multiple regression model, the three predictors together explained 59.1% of the variation in HSB. The HSB was highest in stores with high SEP, in the French-speaking region and in urban areas (Table 4). The effects of SEP and language region partially compensated each other, as stores in the German-speaking region had a higher mean SEP yet a lower HSB than stores in the two other regions (Figure 3A). In contrast, the effects of SEP and urbanicity on the HSB were partially redundant, as stores in rural areas had a lower mean SEP and accordingly, a lower HSB than those in urban areas (Figure 3B). These associations between predictors caused regression coefficients for language regions to be larger and those for urbanicity to be smaller in a multiple regression than in simple regressions (Table 4).

The positions of stores along the healthy-to-unhealthy gradient as revealed by PCA axis 1 depended on SEP, language region and urbanicity in an analogous way to the HSB (Figure S2 in Supplementary Materials), with 66.4% of the variation in PCA axis 1 explained by the three predictors jointly. The “vegetarian-to-meat” gradient (PCA axis 2) showed an even stronger dependence on the three factors, with 75.1% of the variation explained. Stores in low-SEP neighborhoods, in the French-speaking region and in rural areas sold more meat and fish (Figure S2). The positions of stores along the “low-to-high carbohydrates” gradient (PCA axis 3) were only weakly related to SEP, language region and urbanicity (Figure S2), with only 17.2% of the variation being explained by the three predictors together.

### 3.3. Factors Influencing the Mean BMI of Conscripts

**Figure 4:**
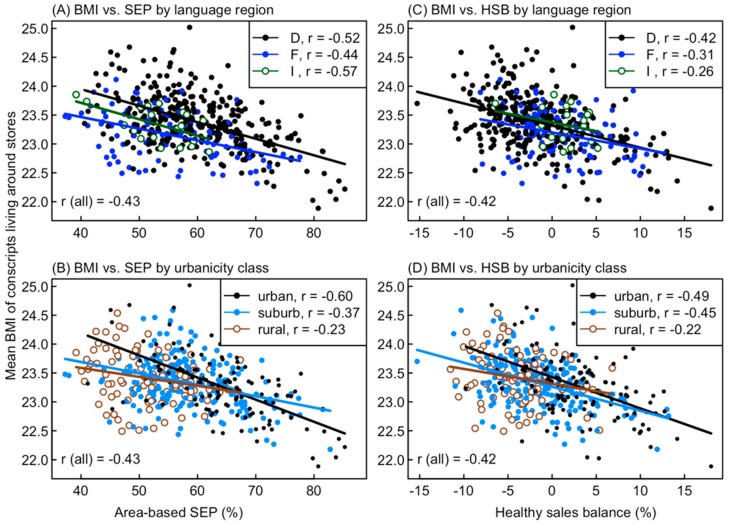
Mean BMI of conscripts living around the 445 stores in relation to the (**A**,**B**) area-based SEP and (**C**,**D**) the healthy sales balance (HSB) of the stores, either by language region (D = German, F = French, I = Italian) or by urbanicity class. Pearson correlation coefficients and ordinary regression lines are given for each subset of stores, and correlation coefficients are also given for all stores together. Confidence intervals for correlation coefficients are given in Table S1.

The mean BMI of conscripts was negatively related to the area-based SEP (*r* = −0.43, Figure 4A,B) and HSB (*r* = −0.42, Figure 4C,D). The relationship between BMI and SEP was modified by language regions and urbanicity, as the mean BMI was higher in the German region than in the French region (Figure 4A), and showed a weaker dependence on the SEP (flatter regression line) in rural areas than in urban or suburban ones (Figure 4B). The dependence of the mean BMI on the HSB was consistent across the three language regions, which had almost identical regression lines (Figure 4C), whereas it was weaker (flatter regression line) in rural areas than in urban or suburban ones (Figure 4D). Other measures of population-level weight status showed similar but slightly weaker correlations with the HSB, e.g., median BMI (*r* = −0.36) or the percentage of conscripts with BMI > 25 (*r* = −0.39). In a simple regression model, the mean BMI decreased by 0.47 kg/m^2^ from the lowest to the highest quartile of HSB, but this difference almost vanished (0.07 kg/m^2^) after adjusting for SEP, language region and urbanicity in a multiple regression (Table 5).

### 3.4. Spatial Patterns

**Figure 5:**
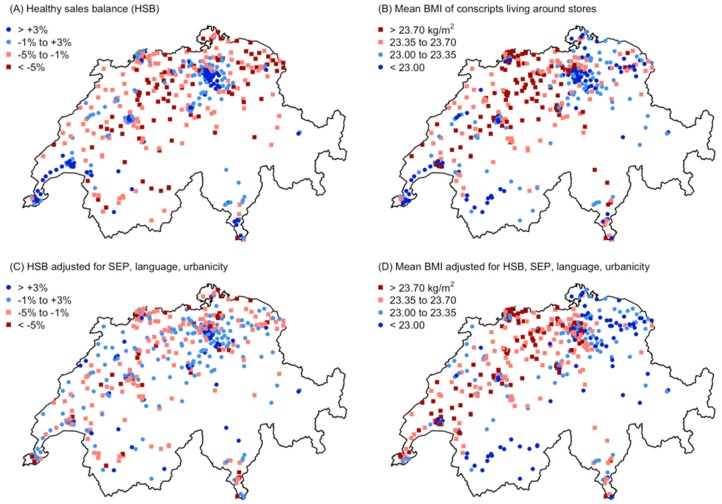
Spatial distribution of the healthy sales balance of the 445 stores and the mean BMI of conscripts living around the stores, either as raw data (**A**,**B**) or adjusted for the predictors in multiple regression models (**C**,**D**). Symbol colors represent values of HSB or BMI in four categories that approximately correspond to quartiles. Blue indicates healthy conditions (high HSB, low BMI). The spatial pattern in HSB (**A**) was largely accounted for by the regression model, so adjusted values show little spatial structure (**C**). In contrast, the spatial pattern in BMI (**B**) was poorly accounted for by the regression model, so adjusted values still show a pronounced spatial structure at the regional scale (**D**).

Both the HSB and the mean BMI showed a clear regional pattern across Switzerland, but there was also strong small-scale variation within cities (Figure 5A,B). The spatial pattern in HSB was largely accounted for by the multiple regression model, so that adjusted HSB values showed little spatial structure (Figure 5C). In contrast, the spatial pattern in mean BMI was poorly accounted for by the regression model, so that adjusted BMI values still showed pronounced regional differences, with high BMI values in northwestern Switzerland and low BMI values in northeastern Switzerland and Alpine regions (Figure 5D). This result did not depend on the specific regression model used, as alternative models (without HSB; including interaction terms; including the food sales PCA axis 1) revealed the same spatial pattern of adjusted mean BMI (Figure S3 in Supplementary Materials). Thus, regional differences in BMI that were not accounted for by SEP, language region or urbanicity [22] could not be explained by differences in the store-level food sales composition.

Consistent with spatial patterns in adjusted values, the inclusion of a spatial correlation structure in the multiple regression for HSB (Table 4) did not have any effect on regression coefficients and confidence intervals (not shown). In contrast, some regression coefficients for mean BMI (Table 5) changed. In particular, the effect of urbanicity vanished: Differences between urban centers and rural areas varied regionally and were therefore better represented by a regional spatial structure, as is apparent in Figure 5D.

## 4. Discussion

### 4.1. Strong Variation in Sales of Healthy and Unhealthy Food Types

Our analysis shows that the percentage sales of different food groups commonly regarded as healthy or unhealthy vary strongly among stores of a single large supermarket chain. These variations were mutually associated so that stores could be ranked along a continuum from a more healthy to a more unhealthy sales pattern. Even if correlations between individual food types were weak to moderate, the sum of healthy food sales varied three-fold among stores, and the sum of unhealthy food sales varied almost two-fold. Thus, despite their “healthful” food offer [15,16], large supermarkets do not always sell a high proportion of healthy foods. Large variation in the “healthfulness” of food purchases in supermarkets has also been demonstrated elsewhere based on data from customer cards, till receipts [20,37] or purchase diaries [17,38]. Variation in the “healthfulness” of food offers was demonstrated using metrics such as product variety, price and quality [16] or the space allocated to healthy and unhealthy food types within stores [39]. Our study expands on previous research by considering actual sales (rather than offer) using objective and precise records (without self-reporting bias) with full spatial coverage (an entire country) over an extended time period (one year) and including a broad range of food items (not only clearly healthy and unhealthy ones).

The large variation among stores found here is remarkable for several reasons. First, Switzerland has a high overall living standard and a policy of limiting area-based social differences [40], so the prevalence and spatial clustering of poverty is moderate relative to most other countries. Second, the supermarket chain investigated here has an intermediate price level and does not sell alcohol or tobacco, suggesting some bias towards more affluent and health-oriented customers compared with discount supermarkets. Third, short distances between stores and good public transport mean that stores can recruit their customers from wider, overlapping spatial ranges rather than just the nearest postcodes assigned to them in this study. Finally, all stores of the supermarket chain have a similar in-store setting, with a prominent market-like display of fresh fruit and vegetables at the store entrance and less prominent displays of typical unhealthy food types. Without these equalizing factors, differences among stores might be even more pronounced.

We summarized variation among stores by the HSB, i.e., the difference between the percentage of sales from healthy and unhealthy food types. Alternatively, Howard Wilsher et al. [18] used the “unhealthy sales percentage” (unhealthy sales relative to the sum of healthy and unhealthy sales), and Thiele et al. [17] used the first axis of a principal components analysis (PCA) of detailed food purchase data. In our data, the HSB correlated almost perfectly with the “unhealthy sales percentage” (*r* = −0.998), and it correlated strongly with the first PCA axis (*r* = −0.945), so results similar to those presented here were obtained with the two alternative measures. We preferred the HSB because differences between healthy and unhealthy food sales are expressed relative to total food sales, which indicates their importance in the overall diet.

The strong negative correlation between healthy and unhealthy percentage sales (Figure 2) may suggest a trade-off or deliberate choice between purchases of these food types. However, we must consider that increased purchases of healthy foods automatically reduce the percentage sales of all other foods, even if amounts purchased remain identical. To evaluate the resulting bias, we randomly permuted healthy percentage sales among stores while keeping all other sales fixed, re-adjusted percentages to a sum of 100% and then computed the correlation. After 1000 runs, the mean correlation between randomly associated healthy and unhealthy percentages was −0.57. The actual correlation (−0.85) was more negative, indicating some trade-off or deliberate choice, but not to the point suggested by Figure 2. This concurs with individual-level surveys showing, e.g., that different health attitudes cause large differences in fruit and vegetable consumption but only small differences in the energy density of the diet (e.g., [41]). Experiments further showed that interventions to promote the purchase of fruit or vegetables mostly succeed in this respect but do not necessarily reduce purchases of less healthy foods or consumers’ energy intake [42], which limits their effectiveness in obesity prevention.

Some exceptions in the general pattern of healthy vs. unhealthy food sales were remarkable: Sales of red meat, which is generally consumed more than recommended for health and sustainability in Switzerland [6], and sales of chocolate and cookies were only weakly associated with this pattern, i.e., not more than sales of bread (Table 3). A more detailed analysis showed that sales of red meat correlated positively with sales of unhealthy food types within each language region, but negatively between language regions—more red meat was sold in the otherwise “healthier” French-speaking region (results not shown). These opposite associations within and between language regions just compensated each other. Chocolate consumption is generally high in Switzerland, which may create a social norm that overrides concerns about its high energy density [43,44]. Similarly, fruit juices and breakfast cereals were associated with the healthy food pattern despite their sugar content. These food types are commonly assumed to be part of a healthy lifestyle, and this image seems to be more relevant for consumers’ behavior than the actual nutritional value [45].

### 4.2. Environmental Determinants of Food Sales Patterns

The positive correlation found here between the HSB and the area-based SEP may seem to support the view that a healthy yet palatable and culturally acceptable diet is unaffordable for low-income consumers [46], or that an unfavorable composition of food outlets in deprived neighborhoods promotes unhealthy food sales [47]. However, even if the real or perceived higher price of healthy foods does influence less healthy food choices [17], several other factors were found to be equally important, especially in high-income European countries, including education and health attitudes [15,17,41,48,49], taste preferences and convenience [37,38,50] as well as social or cultural influences [43,51]. Among customers of the supermarket chain studied here, inability to afford healthy food was most likely rare given the low poverty rate in Switzerland and the presence of cheaper discount supermarkets in urban and many suburban areas; the lowest HSB values were found in some rural and suburban areas (Figure 3A), where cheaper alternatives may have been missing. The Swiss area-based SEP index actually combines information on income, education and occupation [34], and all three components may contribute to the association between SEP and HSB. Education influences health attitudes and motivation [37,41,52], while occupation influences daily energy requirements and dietary norms [53].

The HSB and underlying food sales patterns also differed between language regions. The higher HSB found for the French region—especially after adjusting for differences in SEP—was due to greater sales of fish, slightly greater sales of vegetables and fewer sales of processed meat (sausages and cold meat) and sweet drinks. The same patterns were found using detailed individual-level food consumption data in the National Nutrition Survey (menuCH) [6] and the Swiss Health Survey 2012 [52]. Our sales data and menuCH data [6] also both showed a higher consumption of unprocessed red meat in the French region. This further highlights the influence of sociocultural factors as opposed to purely financial ones on the choice of a more or less healthy diet. 

### 4.3. Association of Mean BMI with Food Sales Patterns

Swiss Army conscripts living around stores with healthy food sales had, on average, lower BMI values. This supports our assumption that food sales of a single supermarket chain are indicative of environmental factors that influence the BMI of young men. Yet, the effect size was modest and almost vanished after adjusting for confounding social factors (Table 5). The percentage of overweight or obese conscripts decreased from 26.2% to 22.0% between the lowest and highest quartiles of HSB, which is similar to the 3.5% difference in overweight/obese British school children found by Howard Wilsher et al. [18] between the two extreme quartiles of unhealthy food sales percentage. However, after adjusting for SEP, language regions and urbanicity, the difference vanished (0.6%) in our data, whereas Howard Wilsher et al. [18] still found a 2.7% difference.

The fact that differences in BMI among HSB quartiles vanished after adjusting for SEP, language region and urbanicity suggests that the association between mean BMI values and store-level HSB was partially indirect, reflecting more general socioeconomic characteristics of the conscripts’ neighborhoods. In support of this interpretation, the food category most strongly correlated with the BMI was organically produced food (Figure S1), although this category includes items spanning the full range of energy density and cannot by itself contribute to weight control. The purchase of such items rather reflects a general interest in health and sustainability [54], which is more prevalent among customers with high education and income [52].

The rather weak association of mean BMI with store-level HSB cannot be attributed to low data quality: The Army’s recruitment procedure provided us with objective, precisely measured BMI data for a sample of high coverage from a well-defined population [23]. However, the use of army conscription data is a limitation of our study, as it restricted our BMI data to young men with Swiss citizenship, whereas food sales data reflect purchases made by people of any age, place of residence and nationality. Thus, we compared spatial variations for different subpopulations. The mean BMI value of young men measured in this study certainly correlates with that of the older adult population, including women, because overweight adolescents usually have overweight parents and rarely lose their excess weight in later life [55]. However, some young men have a low BMI value despite an energy-dense diet due to rapid height growth in late adolescence, and sporty young men may have an elevated BMI due to having a high muscle mass rather than high fat content [56]. For both reasons, our results probably underestimated the association of mean BMI with store-level HSB expected to occur across the entire population. In regard to the possible influence of non-residents and foreigners, we checked that stores located in railway stations or major touristic resorts did not have a particularly high or low HSB. We also found that the correlation between mean BMI and store-level HSB did not differ between municipalities with more or less than the median proportion (22%) of foreign residents (*r* = −0.42 in both data subsets), suggesting that this correlation was not affected by the exclusion of foreigners from our BMI data. 

Further limitations of our study that may have played a role are as follows: First, our food sales data only covered one supermarket chain in Switzerland, whose stores are distributed heterogeneously across the country (Figure 1). The second largest supermarket chain is similar in product offer, price level and selling concepts, but discount supermarkets and convenience stores typically have a more unhealthy sales pattern [15], so some variation in BMI may be related to the differential use of such alternatives by the conscripts or their parents. Second, we matched conscripts with stores based on spatial proximity, which is only one of the many factors that influence customers’ choice of food outlets [57]. Conscripts (or their parents) may have purchased most of their food at other suitable locations, such as their place of work or study. Third, the supermarket chain studied here does not sell alcohol or tobacco. Variation in BMI related to alcohol consumption and smoking (including parental smoking) could therefore not be represented. This might also explain why salty snacks (often consumed together with alcohol) represented only 0.6% of total sales (Table 2) and were hardly related to the mean BMI (Figure S1). Fourth, store-level food sales data can only be analyzed in relative terms (sales composition) and do not provide direct information about individual-level food consumption and energy intake. However, other studies consistently found more unhealthy nutrition patterns to be associated with higher daily energy intake [58]. Finally, we did not consider variation in physical activity, media use and other life habits that can be related to excess weight in adolescents and adults [2,4].

Most importantly, this was a cross-sectional, population-level analysis. Recent individual-level surveys, which were not affected by the above limitations, still showed weak cross-sectional associations between “healthy/unhealthy” nutrition patterns and weight status or fat mass after accounting for important confounders such as parent BMI [59], mother’s education [49] or income and occupation [58]. In contrast, longitudinal studies showed that increases in an individual’s BMI and fat mass during childhood and adolescence are related to unhealthy dietary patterns [58,60] and to the consumption frequency of unhealthy foods (e.g., [61]). 

## 5. Conclusions

Our study confirmed that food sales patterns among stores of a large supermarket chain may serve as an indicator of spatial variation in dietary patterns [18,19,20]. The automatic availability, objectivity, precision and anonymity of store-level sales data allows dietary patterns to be studied at a scale and level of resolution that are typically not feasible with individual-level surveys.

Store-level food sales data could be used to design interventions for healthier nutrition and to monitor their effect at the population level over time [18]. For example, pre-existing differences in healthy vs. unhealthy food sales may serve as a selection criterion or as stratifying factor when selecting a sample of stores for an intervention. In doing so, cultural differences (e.g., different language regions) may have to be taken into account. These baseline differences should also be taken into account in the evaluation of intervention effects. Furthermore, the most effective type of intervention for a particular area (e.g., health information, social norm messages or pricing incentives) may depend on the initial HSB [45,62].

Our study confirmed the general associations between low SEP, unhealthy diet and prevalence of excess weight found worldwide [1], but these cross-sectional associations seemed to be mostly indirect. It remains unclear whether an improved balance of healthy food sales will really lead to a decrease in excess weight at population level [59]. Our approach could be used in further studies to analyze whether changes in the mean HSB of stores over time are associated with changes in the BMI of conscripts or other representative population groups living in the stores’ neighborhoods. 

## Figures and Tables

**Table 1:** Characteristics of the stores included in the data analysis: number (*n*) of stores per language region and urbanicity class as well as means ± SD of the area-based socioeconomic position (SEP, on a scale from 0 to 100) and the mean Body Mass Index (BMI) of conscripts attributed to each store.

Factor	Category	*n*	Means ± SD among Stores	
			Area-Based SEP	Mean BMI (kg/m^2^)
Overall	all	445	58.45 ± 8.90	23.33 ± 0.46
Language region (2013)	German (D)	324	59.93 ± 8.60	23.38 ± 0.48
	French (F)	101	54.71 ± 8.89	23.17 ± 0.41
	Italian (I)	20	53.40 ± 6.12	23.33 ± 0.31
Urbanicity class (DEGURBA, Eurostat 2011)	urban	120	64.12 ± 8.86	23.26 ± 0.56
	suburban	232	58.43 ± 7.59	23.33 ± 0.41
	rural	93	51.20 ± 6.39	23.41 ± 0.44

**Table 2:** Food types included in our analysis including their assignment to three categories of nutritional value and the distribution of their sales percentage (annual sales volume as % of total sales per store) among the 445 stores.

Food Type	Category and Rationale H = Healthy, U = Unhealthy, B = Both H&U or Basic Food	Mean ± SD (% of Total)	Range (% of Total)
Fruit	H: vitamins and minerals	6.77 ± 0.95	3.8–10.0
Vegetables, fresh and preserved	H: low energy, fiber, vitamins and minerals	7.72 ± 0.98	3.9–10.8
Legumes	H: proteins, fiber, minerals	0.12 ± 0.05	0.0–0.3
Fish	H: protein, relatively low fat	1.87 ± 0.79	0.8–5.1
Food supplements ^1^	H: “healthy lifestyle” product	0.24 ± 0.07	0.0–0.5
Organically produced food ^2^	H: “healthy lifestyle” product	3.65 ± 1.53	1.2–11.5
Crisps	U: high fat and salt content	0.63 ± 0.13	0.4–1.2
Sausages and cold meat	U: high fat and salt content	10.12 ± 1.31	6.2–13.6
Sweet drinks	U: high sugar content	1.81 ± 0.52	0.8–4.3
Ice-cream	U: high sugar and fat content	1.38 ± 0.25	0.6–2.2
Chocolate and cookies ^3^	U: high sugar and fat content	3.61 ± 0.71	2.2–8.7
Cakes	U: high sugar and fat content	3.66 ± 0.64	1.5–6.1
Fruit juice	B: both: vitamins/high sugar	1.02 ± 0.16	0.6–1.6
Breakfast cereals	B: both: “healthy lifestyle” but partly high sugar ^4^	1.17 ± 0.19	0.7–2.4
Bread	B: basic food	5.27 ± 0.95	3.2–8.8
Pasta	B: basic food	1.22 ± 0.18	0.8–2.8
Meat (beef, pork, lamb)	B: basic food for non-vegetarians, partly high fat	5.78 ± 1.08	2.6–9.0
Poultry	B: basic food for non-vegetarians	3.44 ± 0.63	1.9–5.2
Eggs	B: basic food	1.43 ± 0.19	0.8–2.1
Milk and dairy products	B: basic food, partly high sugar/fat ^4^	16.16 ± 1.23	11.9–19.4
Total	All classes above without organic food	73.41 ± 2.15	63.8–78.0

^1^ e.g., vitamins, minerals, sport foods and supplements, weight control products. ^2^ This food type was non-exclusive of the others, i.e., food items could be included in this type and another food type. ^3^ Excluding Christmas/Easter chocolates and pralines. ^4^ The available classification did not allow a subdivision into “healthy” and “unhealthy” according to sugar and/or fat content.

**Table 3:** Loadings of each food type on the first three axes of a principal components analysis (PCA) based on a correlation matrix of sales percentages. The fraction of variation represented by each PCA axis is given in the first row. For the first PCA axis, light-grey shading indicates food types associated with the healthy side of the sales gradient, and dark-grey shading indicates the food types associated with the unhealthy side of the sales gradient. Horizontal lines subdivide the three a priori categories (healthy, unhealthy, both/basic, cf. Table 2).

Variation Represented	Axis 1	Axis 2	Axis 3
	25.4%	16.7%	12.3%
Fruit	−0.35	−0.16	−0.04
Vegetables	−0.34	−0.14	−0.24
Legumes	−0.25	0.34	0.19
Fish	−0.31	0.32	0.10
Food supplements	−0.14	−0.10	−0.17
Organic food	−0.26	−0.33	−0.06
Crisps	0.31	0.06	−0.08
Sausages and cold meat	0.33	0.18	−0.28
Sweet drinks	0.24	−0.28	0.12
Ice-cream	0.23	−0.08	−0.20
Chocolate and cookies	0.08	0.12	0.43
Cakes	0.18	−0.07	0.36
Fruit juices	−0.18	0.01	0.12
Breakfast cereals	−0.10	−0.09	−0.14
Bread	0.07	−0.31	0.34
Pasta	0.17	0.08	0.18
Meat	0.08	0.36	−0.24
Poultry	−0.15	0.37	−0.09
Eggs	−0.11	−0.32	−0.11
Milk and dairy products	0.21	−0.09	−0.38

**Table 4:** Coefficients from regression models for the effects of area-based SEP (socioeconomic position, categorized into quartiles), language region and urbanicity on the healthy sales balance (difference between healthy and unhealthy sales as % of total food sales) with 95% CI. Simple regression means that separate models were fitted for each predictor, while the multiple regression included all predictors in one model. Each predictor’s reference category is included in the table with a coefficient of 0 for clarity.

Predictor Regression	Simple Regression		Multiple	
Area-based SEP quartiles				
low	0.0	-	0.0	-
low-mid	0.9	(−0.2 to 2.0)	1.3	(0.3 to 2.3)
mid-high	3.0	(1.9 to 4.2)	3.1	(2.1 to 4.2)
high	7.6	(6.5 to 8.7)	7.5	(6.4 to 8.6)
Language				
German	0.0	-	0.0	-
French	2.6	(1.5 to 3.8)	4.0	(3.2 to 4.9)
Italian	3.0	(0.7 to 5.2)	5.2	(3.5 to 6.8)
Urbanicity				
rural	0.0	-	0.0	-
suburban	2.4	(1.3 to 3.5)	1.1	(0.2 to 2.1)
urban	7.0	(5.7 to 8.2)	3.6	(2.4 to 4.7)

**Table 5:** Coefficients (with 95% CI) from regression models for the effect of the healthy sales balance (HSB), categorized into quartiles, on the mean BMI of conscripts. The multiple regression model included the area-based SEP (socioeconomic position), language region and urbanicity as additional predictors to adjust for their confounding effects. The lowest HSB quartile is included in the table with a coefficient of 0 for clarity.

Predictor Regression	Simple Regression		Multiple	
low	0.0	-	0.0	-
low-mid	−0.14	(−0.25 to −0.03)	−0.07	(−0.18 to 0.05)
mid-high	−0.38	(−0.40 to −0.17)	−0.13	(−0.25 to 0.00)
high	−0.47	(−0.58 to −0.35)	−0.19	(−0.34 to −0.04)

## Supplementary Materials

The following are available online at https://www.mdpi.com/2072-6643/11/3/579/s1. Figure S1: Correlations between sales of 20 food types, area-based socio-economic position (SEP), and mean BMI of conscripts across the 445 stores: Pearson correlation coefficients for all pairs of variables are given as numbers above the diagonal and visualized by colours below the diagonal, Figure S2: Distribution of the scores of 445 stores on PCA axis 1, 2 and 3, in relation to the area-based SEP (in quartiles), language region and urbanicity class, Figure S3: Spatial distribution of the mean BMI of conscripts, adjusted for predictors according to three models that differed from the multiple regression in the main part of the paper (Table 4, Figure 5D) as follows: (A) HSB not included in model; (B) all two- and three-way interaction terms includ-ed in model; (C) PCA axis 1 (healthy-unhealthy gradient) included in model instead of the HSB.
