# Supplementary material for: Spatial Association of Food Sales in Supermarkets with the Mean BMI of Young Men: An Ecological Study
Source: Nutrients. 2019 Mar 8;11(3):579. doi: 10.3390/nu11030579 (PMC6470871; doi:10.3390/nu11030579)

Supplementary Material

Figure S1. Correlations between sales of 20 food types, area-based socio-economic position (SEP), and mean BMI of conscripts across the 445 stores: Pearson correlation coefficients for all pairs of variables are given as numbers above the diagonal and visualized by colours below the diagonal. Subdivisions indicate the a-priori classification of food types (see Table 2 in main document).

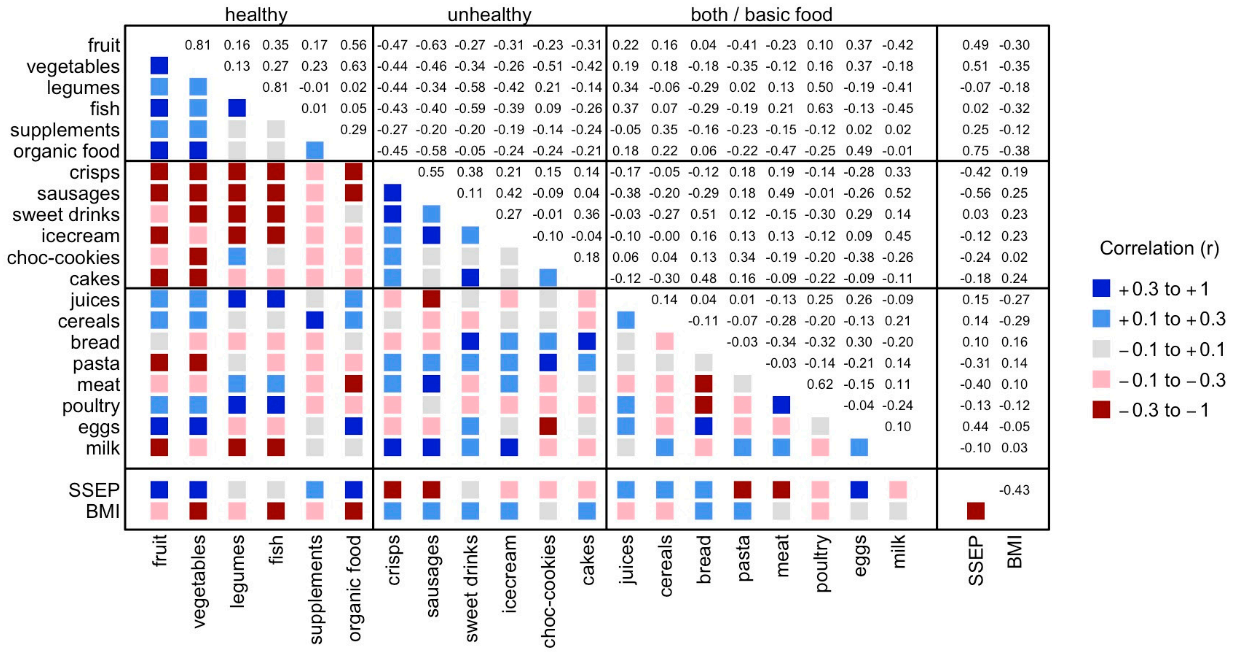

Figure S2. Distribution of the scores of 445 stores on PCA axis 1, 2 and 3, in relation to the area-based SEP (in quartiles), language region and urbanicity class.

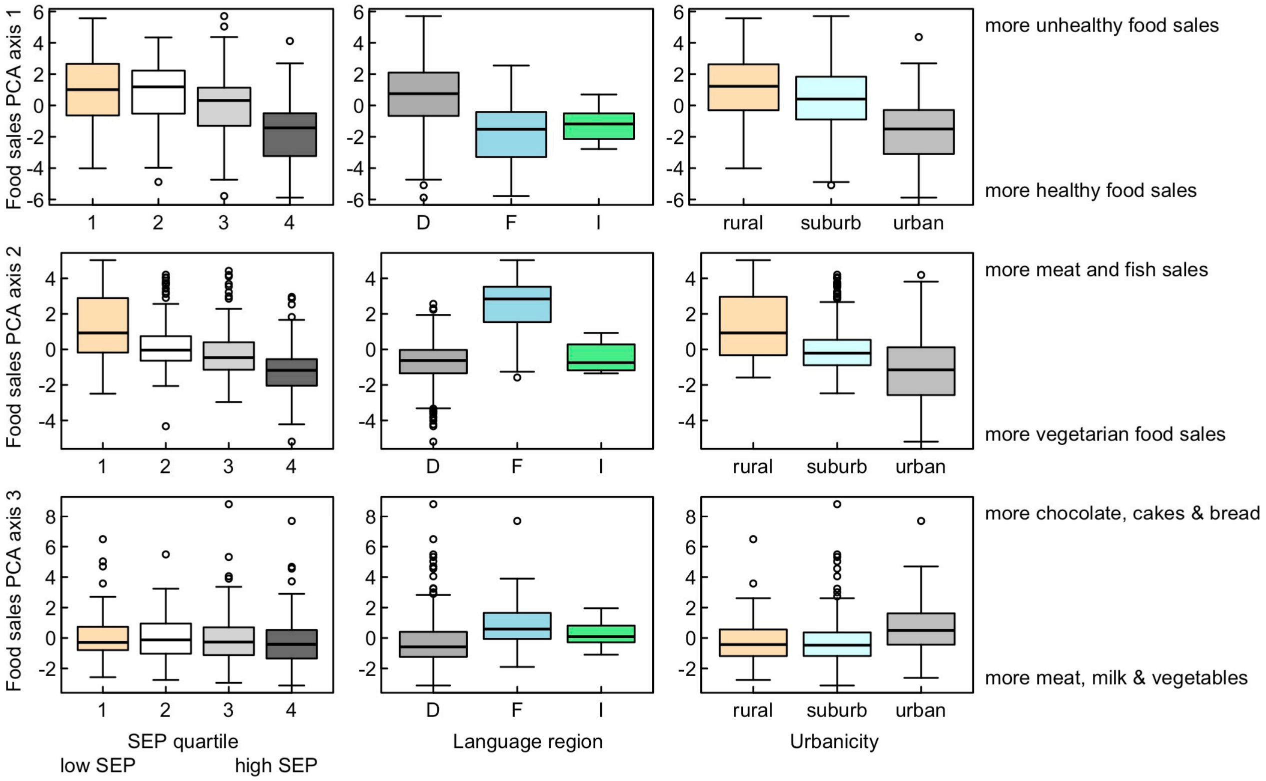

Figure S3. Spatial distribution of the mean BMI of conscripts, adjusted for predictors according to three models that differed from the multiple regression in the main part of the paper (Table 4, Figure 5D) as follows: (A) HSB not included in model; (B) all two- and three-way interaction terms included in model; (C) PCA axis 1 (healthy-unhealthy gradient) included in model instead of the HSB. Symbol colours represent values of mean BMI in four categories that approximately correspond to quartiles. Maps show that spatial patterns of adjusted values were similar with all models.

(A) BMI adjusted for SEP, language, urbanicity

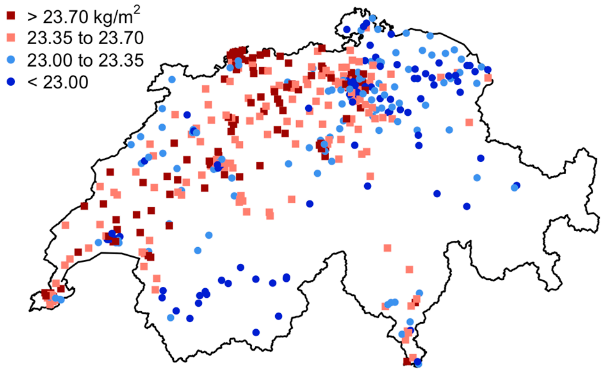

(B) BMI adjusted for HSB, SEP, language, urbanicity and interactions

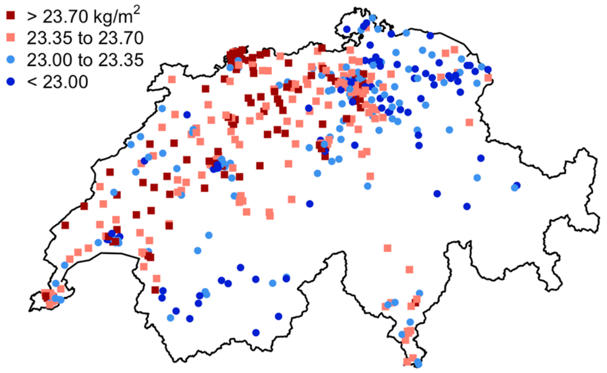

(C) BMI adjusted for food sales PCA axis 1, SEP, language, urbanicity

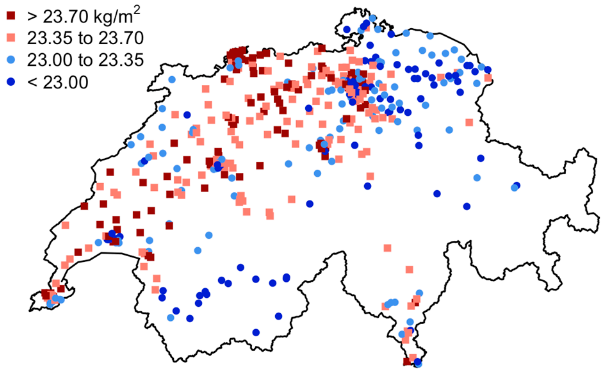

Supplement: Supplementary file 1 [file nutrients-11-00579-s001.pdf]
